# The haemagglutinin gene of bovine-origin H5N1 influenza viruses currently retains receptor-binding and pH-fusion characteristics of avian host phenotype

**DOI:** 10.1080/22221751.2025.2451052

**Published:** 2025-01-13

**Authors:** Jiayun Yang, Mehnaz Qureshi, Reddy Kolli, Thomas P. Peacock, Jean-Remy Sadeyen, Toby Carter, Samuel Richardson, Rebecca Daines, Wendy S. Barclay, Ian H. Brown, Munir Iqbal

**Affiliations:** a The Pirbright Institute, Pirbright, UK; b Department of Infectious Disease, Imperial College London, London, UK

**Keywords:** High pathogenicity avian influenza, cattle and goat H5N1, receptor binding, pH fusion, zoonotic risk

## Abstract

Clade 2.3.4.4b H5N1 high pathogenicity avian influenza virus (HPAIV) has caused a panzootic affecting all continents except Australia, expanding its host range to several mammalian species. In March 2024, H5N1 HPAIV was first detected in dairy cattle and goats in the United States. Over 891 dairy farms across 16 states have tested positive until 25 December 2024, with zoonotic infections reported among dairy workers. This raises concerns about the virus undergoing evolutionary changes in cattle that could enhance its zoonotic potential. The Influenza glycoprotein haemagglutinin (HA) facilitates entry into host cells through receptor binding and pH-induced fusion with cellular membranes. Adaptive changes in HA modulate virus-host cell interactions. This study compared the HA genes of cattle and goat H5N1 viruses with the dominant avian-origin clade 2.3.4.4b H5N1 in the United Kingdom, focusing on receptor binding, pH fusion, and thermostability. All the tested H5N1 viruses showed binding exclusively to avian-like receptors, with a pH fusion of 5.9, outside the pH range associated with efficient human airborne transmissibility (pH 5.0–5.5). We further investigated the impact of emerging HA substitutions seen in the ongoing cattle outbreaks, but saw little phenotypic difference, with continued exclusive binding to avian-like receptor analogues and pHs of fusion above 5.8. This suggests that the HA genes from the cattle and goat outbreaks do not pose an enhanced threat compared to circulating avian viruses. However, given the rapid evolution of H5 viruses, continuous monitoring and updated risk assessments remain essential to understanding virus zoonotic and pandemic risks.

## GRAPHICAL ABSTRACT

**Figure:**
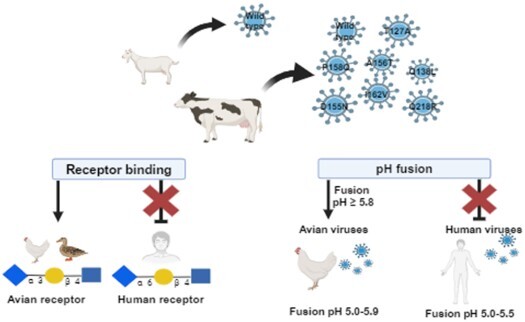

## Introduction

Influenza A virus (IAV) infection is a major global disease that continues to threaten wildlife, poultry production and human health. IAV is a negative-sense, single-stranded stranded and segmented RNA virus. Wild aquatic birds act as the natural reservoirs of IAV, harbouring 17 HA (H1-H16 and H19) and 11 NA subtypes [1, 2]. The global poultry industry has been severely impacted by the wild bird-driven spread of Avian influenza virus (AIV). Since the first detection of high pathogenicity avian influenza virus (HPAIV) H5 subtype in 1996, its precursor A/goose/Guangdong/1/96 (Gs/Gd/96) lineage has then rapidly evolved into more than 30 subclades [3, 4]. The current clade 2.3.4.4b H5N1 has become dominant since 2021 with noticeable genotypic change from predominant H5N8 to H5N1, which then spread globally[5]. Panzootic H5N1 viruses have then caused numerous outbreaks in avian species worldwide with unprecedented infections. In addition to birds, the clade 2.3.4.4b H5N1 viruses have also caused several outbreaks in mammalian species, such as in minks, foxes, domestic cats, and semi-aquatic mammals [6–9]. Human infection of HPAIV H5N1 remains sporadic. There have been 903 cases of human infections resulting in 464 deaths since 2003 [10], but only several dozen of these viruses have occurred in the recent panzootic.

However, with the increasing reports of clade 2.3.4.4b H5N1 infections in mammalian species, it is a concern that the virus could adapt to transmit efficiently by the airborne route which might facilitate infection of humans. While there were no reports of H5N1 virus infection in livestock like goats and cattle until March 2024, the Minnesota Board of Animal Health (MBAH) reported fatal infections of clade 2.3.4.4b H5N1 virus in newborn goats in February following the culling of HPAIV poultry housed on the same premises. The transmission may have been caused by shared husbandry and water sources [11]. On 25 March 2024 United States Department of Agriculture (USDA) reported detection of clade 2.3.4.4b H5N1 virus in milk samples collected from symptomatic dairy cattle in Kansas and Texas [12]. A dairy farm worker with conjunctivitis was also found to be infected by a clade 2.3.4.4b H5N1 virus [13]. As of 25 December 2024, the H5N1 virus has been further detected in cattle in sixteen states across the US in 891 farms. At present, the virus is thought to be predominantly transmitted through milking, and movement of infected animals or contaminated equipment between farms or states [14–16]. The virus transmission route to cattle has been proposed as mechanical resulting in mammary gland infection [17]. All these cases have been attributed to a single genotype B3.13 of clade 2.3.4.4b viruses, which so far has only been detected in the U.S. [18]. In addition, based on Centers for Disease Control and Prevention (CDC) reports, there have been a further 58 human cases, all with mild symptoms but either attributed to contact with infected dairy cattle (*n* = 35) or to poultry (*n* = 21) or to unknown sources in California and Missouri (n = 2) that had acquired infection from dairy cattle and therefore are also “bovine” origin viruses (last accessed 10th December 2024). The outbreaks in livestock and humans highlight the urgent and ongoing need for risk assessment of the virus for potential spillover to humans.

The binding of viral HA to host cell surface sialic acid (SA) containing glycans is a prerequisite for AIV entry into host cells [19], as well as a determinant of host tropism. Influenza viruses from avian species generally have a preference towards α2,3 – (SAα-2,3Gal)-linked SA, whereas human seasonal and pandemic influenza viruses have a preference towards α2,6-(SAα-2,6Gal) linked SA [20], this preference reflects the predominant SA linkages found in the avian gastrointestinal and human upper respiratory tract, respectively [21]. AIVs can switch receptor binding specificity to overcome host barriers and change host tropism by acquiring mutations in the receptor binding site of HA [22]. Upon the attachment of HA to SA, AIV particles are internalised into endosomes and undergo conformational changes triggered by low pH between 5 and 6 in the maturing endosomes [23]. The acidity stability of HA plays a crucial role in virus transmission and host range. The HA of human transmissible viruses is generally more acid-stable with a pH of ≤5.5 for membrane fusion, whereas many avian viruses have less stable HA proteins that fuse at pHs >5.5 [24]. This is thought to be related to the predominant transmission routes of these viruses, human seasonal viruses transmit by the airborne route and require more stability to survive the harsh microenvironments of aerosol particles and mildly acidic respiratory secretions. In addition to pH stability, thermostability of HA has also been shown to be important for AIV transmission in mammals, and may act as a proxy for pH stability [25].

The recent sustained outbreaks in dairy cattle farms raise credible concerns about the virus evolving to acquire mammalian adaptations and becoming zoonotic or potentially pandemic. HA protein is critical for receptor binding and its pH stability is crucial as an indicator of avian to mammalian host transmission. Therefore, we investigated the phenotype of the HAs of these viruses through receptor binding profiles, thermostability and pH stability comparing goat and dairy cattle H5N1 viruses compared with earlier H5N1 clade 2.3.4.4b viruses that circulated in Europe, as well as the emerging mutations in viruses associated with dairy cattle outbreaks. Our findings provide valuable insights into the ongoing evolution of H5N1 viruses in livestock and their potential veterinary and public health implications.

## Materials and methods

### Ethics statement

All the procedures involving embryonated eggs were undertaken in strict accordance with the guidance and regulations of the UK Home Office under project licence number PP6471846. As part of this process, the work has undergone scrutiny and approval by the animal welfare ethical review board at The Pirbright Institute, incorporating the 3Rs and followed by ARRIVE (Animal Research: Reporting of *in vivo* experiment) guidelines for quality, reproducibility, and translatability of animal studies.

### Cells and viruses

Madin-Darby canine kidney (MDCK) cells, human embryonic kidney 293 T (HEK-293 T) cells, and African green monkey kidney epithelial (Vero) cells were acquired from (CSU) of The Pirbright Institute (TPI). The MDCK cells were the parental MDCK cell line acquired from the European Collection of Authenticated Cell Cultures (Catalogue number 85011435, ECACC). The cells were maintained with Dulbecco's Modified Eagle's medium (DMEM) supplemented with 10% fetal calf serum (FCS) (Gibco) with 5% CO2 at 37 °C. The HA and NA sequences of A/dairy cattle/Texas/24-008749-001-original/2024 (H5N1), A/goat/Minnesota/24-007234-003-original/2024 (H5N1), A/chicken/Scotland/054477/2021 (H5N1) and A/chicken/England/085598/2022 (H5N1) (referred to as TX-Cattle, MN-Goat, Sct477/21 and Eng598/22, respectively) were acquired from GISAID (Table S1) and synthesised by GeneScript and cloned into a bidirectional pHW2000 vector. The polybasic cleavage site of H5 HA PLRERRRKR/GLF was replaced with a monobasic PLGTR/GLF cleavage site for the study to be conducted at Containment Level 2 (CL2) laboratories. The internal segments of the RG viruses were from laboratory-adapted strain A/Puerto Rico/8/1934 (H1N1) (PR8). The viruses were rescued by the previously described protocol [26]. The rescued viruses were propagated in 9- to 10-day-old embryonated hen's eggs.

### Site-directed mutagenesis (SDM)

SDM plasmids were generated using a single-site mutagenesis QuikChange II (Agilent) kit following manufacturer's instructions. The primers (5′ to 3′) were synthesised at Merck.

**Table:** 

Mutation	Sense	Primer (5′ to 3′)
T127A	Forward	gctcacccctagtgatgcttcatgatttggccagg
	Reverse	cctggccaaatcatgaagcatcactaggggtgagc
Q138L	Forward	gcttgtccatacctgggagcaccctcc
	Reverse	ggagggtgctcccaggtatggacaagc
D155N	Forward	atctttattgttgggtatgcattgttctttttgataagccacacc
	Reverse	ggtgtggcttatcaaaaagaacaatgcatacccaacaataaagat
A156T	Forward	aatgtggtgtggcttatcaaaaagaacgatacgtacccaacaataaagata
	Reverse	tatctttattgttgggtacgtatcgttctttttgataagccacaccacatt
P158Q	Forward	gcttatcaaaaagaacgatgcataccaaacaataaagataagctacaataata
	Reverse	tattattgtagcttatctttattgtttggtatgcatcgttctttttgataagc
I162V	Forward	gattagtattattgtagcttacctttattgttgggtatgcatcgttcttt
	Reverse	aaagaacgatgcatacccaacaataaaggtaagctacaataatactaatc
Q218R	Forward	cttccacgttgcccgtttactctggatctagtagctatttttgg
	Reverse	ccaaaaatagctactagatccagagtaaacgggcaacgtggaag

### Bio-layer interferometry (BLI)

Propagated viruses were purified through 30% and 60% sucrose gradient cushion at 27,000 rpm for 2 h at 4℃. The purified viruses were normalised by enzyme-linked immunosorbent assay (ELISA) to a known standard of 100 pmol, quantifying viral nucleoprotein (NP) expression [27]. The standardised 100pmol virus was then incubated with 10 μM oseltamivir carboxylate (Roche) and 10 μM zanamivir (GSK) against avian-like sugar analogue 3SLN and human-like sugar analogue 6SLN, and receptor binding affinity was tested by Octet® R8 system (Sartorius) using streptavidin biosensors (Sartorius). Virus binding affinity was normalised to fractional saturation and the concentrations of sugar loadings [28].

### Antisera preparation

Chicken polyclonal antisera against Sct477/21 and Eng598/22 were prepared as previously described [29]. In brief, the viruses were propagated in embryonated hen's eggs and then inactivated with 0.1% (v/v) β-propiolactone (BPL). The inactivated viruses were then passaged three times in embryonated hen's eggs and checked by haemagglutination assay. The inactivated virus was then concentrated by ultracentrifuge at 27,000 rpm for 2 h. Three-day-old specific pathogen-free (SPF) chickens were inoculated with 1024 haemagglutinating unit (HAU) of concentrated inactivated virus mixed with oil emersion adjuvant (Montanide; Seppic) at an adjuvant: virus 7:3 ratio. A boost dosage was given after 10 days. The inoculated chickens were bled at 18, 25 and 38 days post-inoculation for HI validation.

### Haemagglutinin inhibition (HI) assay

Haemagglutinin inhibition assays were performed following World Health Organisation (WHO) guidelines [30]. Haemagglutinin inhibition (HI) test was carried out by incubating two-fold serially diluted chicken antisera with 4HAU for 1 h, and then adding 1% chicken red blood cells (RBCs). The HI titres were recorded after 30 min incubation at room temperature.

### Virus neutralisation test (VNT)

Chicken antisera was heat-inactivated at 56°C for 30 min and then serially diluted with DMEM. The diluted antisera were incubated with viruses at a concentration of 100 TCID50/mL in a 37°C incubator for 1 h. Monolayered MDCK cells were washed with PBS and infected with the virus-antisera mixture for 1 h at 37°C. After three washes with PBS, the cells were maintained in DMEM supplemented with N-tosyl-L-phenylalanine chloromethyl ketone (TPCK) trypsin for three days. Cells were stained with crystal violet, and neutralisation titres were determined based on the highest dilution of antisera that inhibited cytopathic effects in the monolayered MDCK cells.

### Syncytium formation assays

Syncytium formation assays were performed as previously described [31]. In brief, viruses were titrated in Vero cells by using anti-nucleoprotein (anti-NP) mouse monoclonal antibody and horseradish peroxidase-labelled rabbit anti-mouse immunoglobulins (Dako). Monolayered Vero cells were then incubated with 1.0 multiplicity of infection (MOI) of each virus for 1 h at 37°C, the cells were then washed with PBS and incubated at 37°C for 15 h. The Vero cells were then treated with 3.0μg/mL TPCK trypsin in DMEM for 15 min and followed by incubation with PBS ranging from pH 5.2 to 6.0 at 0.1 pH increment for 5 min. The cells were then maintained with DMEM with 10%FCS for 3 h. The cells were then fixed with acetone: methanol (1:1 ratio) and stained with Giemsa stain (Sigma-Aldrich). Stained cell images were taken by EVOS XL imaging system at 400 µm (Life Technologies). The lowest pH that induced visible syncytium formation is recorded as the pH of fusion with multinucleated cells and indistinct cell membranes. The pH of fusion is highlighted in the red frame.

### Thermostability assay

The thermostability assay was tested as previously described [31]. Viruses were diluted with allantoic fluid and normalised to 32HAU/50 µL. The viruses were then incubated in a Thermal cycler (Bio-Rad) at 50°C, 50.7°C, 51.9°C, 53.8°C, 56.1°C, 58.0°C, 59.2°C, and 60°C and 4°C as a control for 30 min, the HA titres were then determined by Haemagglutination assay.

### Haemagglutinin structure prediction and Weblogo

The crystal structure of H5 HA was acquired from Protein Data Bank (https://www.rcsb.org/) with assession number 4JUL. HA structure modelling and prediction were visualised using SWISS-MODEL and visualised and annotated in PyMol version 4.6. WebLogo was created using WebLogo 3 (https://weblogo.threeplusone.com/) following webpage instructions.

### Statistical analysis

Data were analysed and visualised by GraphPad Prism 10.0 (GraphPad Software, USA). Significance was determined by two-way ANOVA multiple comparisons and Ordinary one-way ANOVA Dunnett's multiple comparisons test. Levels of significance (p) are denoted as: 0.01 < *p* < 0.05 having one asterisk, and 0.001 < *p* < 0.01, 0.0001 < *p* < 0.001 and *p* < 0.0001 having two, three or four asterisks, respectively. *p* > 0.05 was considered not significant.

## Results

### HA genes from cattle and goat H5N1 viruses retain high binding affinity to avian receptors

**Figure 1.**
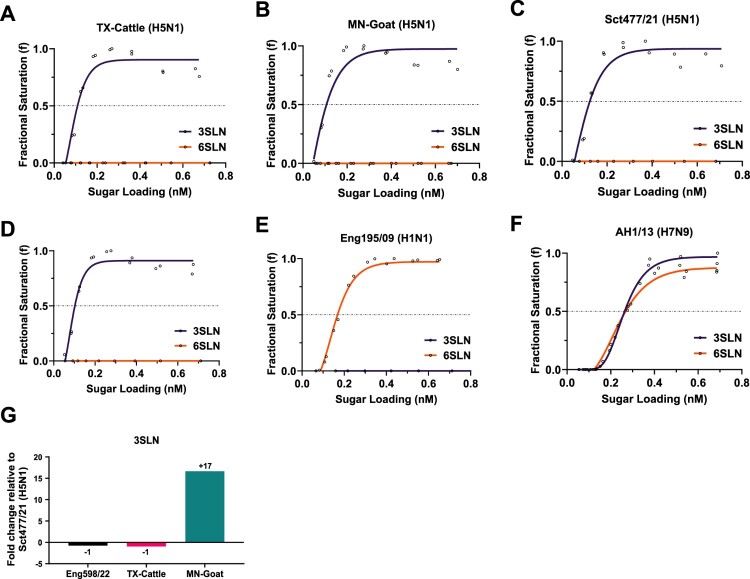
Receptor-binding of the panel clade 2.3.4.4b H5N1 viruses. The receptor binding of the H5N1 viruses was determined by BLI using avian-like receptor analogue 3SLN and human-like receptor analogue 6SLN. (A) TX-Cattle (H5N1), (B) MN-Goat (H5N1), (C) Sct477/21 (H5N1) and (D) Eng598/22 (H5N1), (E) Eng195/09 (H1N1) as assay control, which shows binding to 6SLN only. (F) AH1/13 (H7N9) as assay control, which shows binding to both 3SLN and 6SLN. The fold change of 3SLN relative to Sct477/22 (H5N1) is indicated in (G).

The HA genes from genotype B3.13 associated with early Texas cattle outbreak and Minnesota goat outbreak, TX-Cattle and MN-Goat were selected for comparison with the HA genes of the dominant H5N1 genotypes AIV09 and AIV48 from the UK [32, 33], Sct477/21 and Eng598/22. For each HA, mutagenesis was performed to remove the multibasic cleavage site, and recombinant viruses were rescued by reverse genetics with 6 internal genes from PR8 and homologous NA. Receptor binding profiles against simple sialylated avian receptor analogue α-2,3-sialyllactosamine (3SLN) and human receptor analogue α2,6-sialyllactosamine (6SLN) were assessed by bio-layer interferometry (BLI). Both TX-Cattle and MN-Goat only showed binding affinity to 3SLN without binding to 6SLN (Figure 1A, B). Consistent with our previous study, HA genes of Sct477/21 and Eng085/22 also showed binding to 3SLN only (Figure 1C, D) [34]. A pandemic 2009 H1N1 virus, A/England/195/2009 (referred to as Eng195/09), and a zoonotic H7N9 virus, A/Anhui/1/2013 (H7N9) (referred to as AH1/13) were used as assay controls, with Eng195/09 binding only to 6SLN, and AH1/13 binding to both 3SLN and 6SLN (Figure 1E, F) [35]. The relative binding affinities of the panel viruses to 3SLN were also compared, with Sct477, Eng085/22 and TX-cattle showing comparable binding to 3SLN, while MN-Goat showed 17-fold stronger binding to 3SLN than Sct477/21 (Figure 1G). We also analysed previously reported H5 HA mutations that are related with increased binding affinity to human-type receptors and increased transmission in ferrets [22, 28, 29, 36–38], all amino acids remain “avian” in genotypic characteristics (Table S3).

### HA genes from cattle and goat H5N1 viruses showed membrane fusion at pH 5.9, and goat H5N1 virus showed higher thermostability

**Figure 2.**
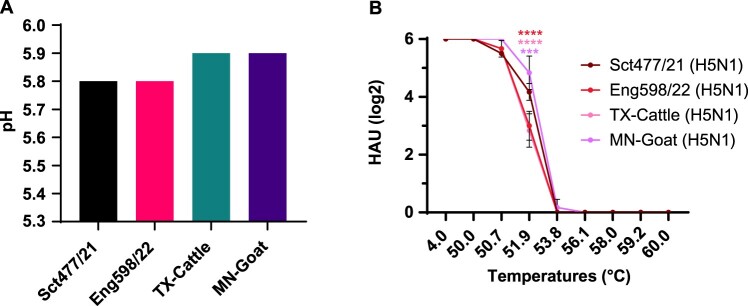
The HA stability of the panel clade 2.3.4.4b H5N1 viruses. (A) pH fusion of the panel H5N1 viruses. Syncytium formation assays were used to evaluate the fusion pH of panel viruses by infecting monolayered Vero cells in a range of pH treatments. (B) Thermostability of the panel H5N1 viruses. Virus loadings were standardised to 64 HAU and then incubated at the indicated temperatures for 30min. Haemagglutination assay was then performed after the incubation. The experiment was performed independently for three times, and levels of significance are shown in the figure, 0.0001 < *P* < 0.001 was given three asterisks and *P* < 0.0001 was given four asterisks.

We then tested the pH that triggered membrane fusion of the virus panel using a syncytium formation assay. Vero cells were infected with the viruses and treated with PBS at different pH values ranging from 5.3 to 6.0. pH of membrane fusion was assigned based on the highest pH at which syncytia formation was observed [38]. Both TX-Cattle and MN-Goat showed syncytium formation at pH 5.9 and below as indicated by red arrows but not at pH 6.0 and Sct477/21 and Eng598/22 at pH 5.8, but not at pH 5.9 (Figure S1). Thus both UK H5N1 viruses were assigned a membrane fusion pH of 5.8, while TX-Cattle and MN-Goat HA fused slightly more readily at pH 5.9 (Figure 2A).

The thermostability of the virus panel was assessed by haemagglutinin assay. The viruses were standardised to 64 hemagglutinin units (HAU) and then heat-treated in a thermocycler from 50°C to 60°C for 30 min or kept at 4°C as a control to test their thermostability. The HAU of the virus panel remained unchanged at 50°C but was completely abrogated at 56.1°C. MN-Goat virus showed the highest thermostability, retaining about 4 times higher HAU at 51.9°C, than TX-Cattle and Eng598/22 (Figure 2B).

### HA genes from cattle and goat H5N1 viruses retained similar antigenicity compared to UK 2.3.4.4b viruses

**Figure 3.**
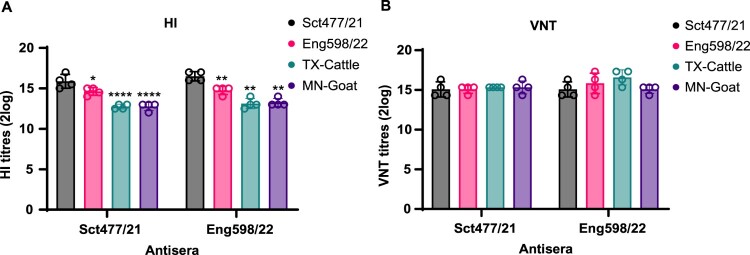
Antigenicity assessment between the panel H5N1 viruses. (A) HI titres of the panel viruses using Sct477/21 and Eng598/22 antisera. HI assays were performed using indicated antisera in four repeats. Levels of significance are shown in figures, 0.01 < *P* < 0.05 was given one asterisk, 0.001<*P*<0.01and *P*<0.0001 were given two, and four asterisks, respectively. *P* > 0.05 was considered not significant. (B) Virus neutralisation of the panel H5N1 virus. Neutralisation tests were performed using the VNT assay with indicated antisera in monolayered MDCK cells for 3 days.

The HA protein is immunodominant and mutations therein modulate antigenicity. We then examined the antigenic differences between the panel of viruses using antisera raised in chickens against Sct477/21 and Eng598/22. TX-Cattle and MN-Goat viruses showed about an 8-fold reduction in HI titres in comparison to homologous titres against Sct477/21 and Eng598/22 (Figure 3A). The antigenicity of the virus panel was also assessed by virus neutralisation test (VNT) in MDCK cells. There was no significant change in virus neutralisation titre (Figure 3B) among the panel viruses (Figure 3B). These data together suggest that the panel of viruses were antigenically similar.

### The emerging HA mutants from cattle H5N1 HPAIV retained binding affinity to avian receptors

**Figure 4.**
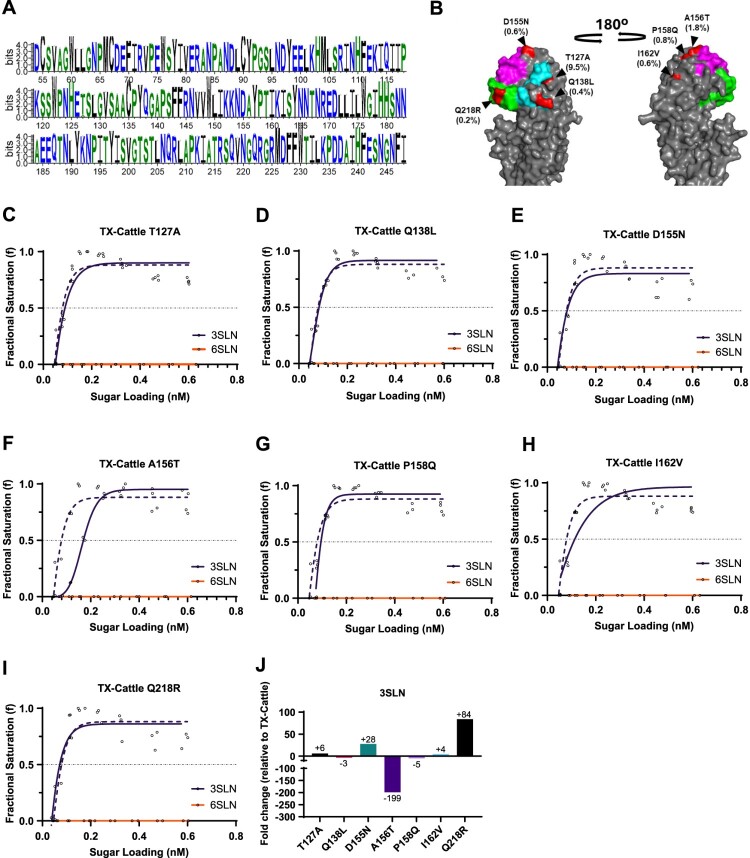
Receptor binding profiles of the emerging mutants from cattle outbreaks. (A) Sequence conservation of the HA globular head region from amino acid position 54 to 248 (H5 numbering) of cattle H5N1 HA sequences. (B) Seven emerging mutations (highlighted in red) are in the HA1 region of HA. Their frequencies (in brackets as percentage) in cattle outbreaks are shown below the mutations. The three key domains of the receptor binding site, 130-loop, 190-helix, and 220-loop, are highlighted in white, green and blue, respectively. BLI was used to assess the receptor binding to avian-like receptor analogue 3SLN and human-like receptor analogue on the following mutant viruses (in solid lines) compared to wild type TX-Cattle (in dotted line): (B) TX-Cattle T143A, (C) TX-Cattle Q154L, (D) TX-Cattle D171N, (E) TX-Cattle A172T, (F) TX-Cattle P174Q, (G) TX-Cattle I178V and (H) TX-Cattle Q234R. The relative fold change of binding affinity to 3SLN relative to the wild type TX-Cattle is shown in (I) with indicated fold change.

Based on 490 available virus sequences uploaded to Sequence Read Archive (SRA), we identified seven emerging mutations in bovine viruses mapping to the HA head domain that could potentially influence receptor binding. These mutations were T127A, Q138L, D155N, A156 T, P158Q, I162 V and Q218R by mature H5 numbering with frequencies of 9.1%, 0.4%, 0.6%, 1.8%, 0.8%, 0.6% and 0.2%, respectively (Figure 4B). We engineered each of these mutations into a recombinant virus using reverse genetics and tested their sialic acid specificity. We found that all the mutant viruses exhibited detectable binding to 3SLN only, but not 6SLN (Figure 4C–I), suggesting the most prevalent mutations in the cattle viruses are not currently resulting in a switch towards human receptor binding. We further compared the fold change in 3SLN binding between mutant viruses to wild-type (WT) TX-Cattle, T127A, D155N, I162 V and Q218R showed increased binding to 3SLN by 6-, 28-, 4- and 84-fold, respectively. Mutants with Q138L, A156 T and P158Q had decreased binding to 3SLN by 3-, 199- and 5-fold, respectively (Figure 4J).

### The emerging HA mutants retained a fusion pH above 5.8, and did not confer any change in thermostability

**Figure 5.**
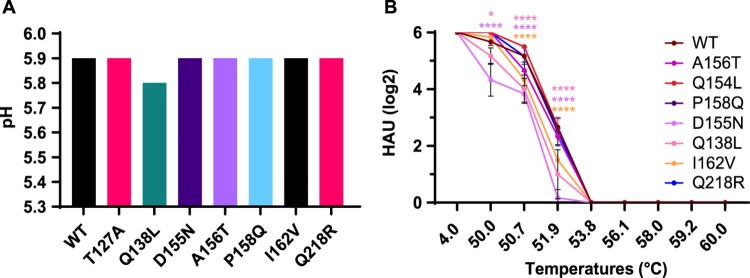
HA stability of the emerging mutants from cattle outbreaks. (A) Required pH for membrane fusion of the mutant viruses. Syncytium formation assays were used to evaluate the fusion pH of mutant viruses by infecting monolayered Vero cells in a range of pH treatments. (B) Thermostability of the H5N1 mutant viruses. Sixty-four HAU of the mutant viruses were heat-treated at the indicated temperatures for 30min. Haemagglutination assay was then performed after the heat-treatment. The experiment was performed independently for three times, and levels of significance are shown in the figure, 0.01 < *P* < 0.05 was given one asterisk and *P* < 0.0001 was given four asterisks.

We tested the pH threshold of membrane fusion for the cattle mutant viruses. Apart from Q138L which did not fuse until pH dropped to pH 5.8, the fusion pH of all six other mutant viruses, T127A, D155N, A156 T, P158Q, I162 V and Q218R, remained unchanged at pH 5.9 compared to the WT TX-Cattle (Figure 5A). The thermostability of the mutant viruses was also tested following heat treatment. A156 T, P158Q, and I162 V showed lower HAU compared to WT at 50.7°C and 51.9°C (Figure 5B), suggesting lower thermostability. All the other four mutations, T127A, Q138L, D155N and Q218R showed comparable stability to WT (Figure 5B).

## Discussion

This study investigated HA properties known to be important in conferring avian to mammalian transmissibility of influenza A viruses including receptor binding, pH of fusion and thermostability in an effort to understand the propensity of the clade 2.3.4.4b virus to cross into cattle and the ongoing risk of zoonotic or pandemic infection in humans. The cattle and goat H5N1 viruses do not carry mammalian adaptations listed in Table S3. Consistent with our BLI data, it showed that the H5 HA of cattle H5N1 HPAIV from Texas virus had receptor binding to avian-receptor analogue 3SLN only. The finding aligns with several studies examining the receptor binding profiles of the human isolate from Texas dairy farm worker and cattle isolate from Ohio, which consistently showed virus binding to avian-type SA receptors only [39–41]. Only one recent study showed that the New Mexico cattle H5N1 virus displayed dual receptor binding to both human and avian receptors by solid-phase ELISA assay [42]. The only amino acid difference between the HA sequences of cattle Texas H5N1 virus and the New Mexico H5N1 virus was at S323N by H5 numbering. This mutation appears unlikely to contribute to changes in receptor binding suggesting the discrepancy could be due to methodological differences. In addition, we also tested the receptor binding of the virus from the Minnesota goat outbreak and the virus also did not show receptor binding to human-receptor analogue 6SLN. Although the polybasic cleavage site was replaced with monobasic cleavage site, previous studies show the replacement is unlikely to change the binding profiles of HPAIV H5N1 HA [28, 36]. Overall, these results indicate that the HA genes from the recent cattle and goat outbreak viruses retained strong binding specificity to avian receptors.

The pH stability and thermostability of the viral HA protein are important for host range and virus transmission. Avian influenza viruses generally have a higher pH of membrane fusion (pH > 5.5) compared to human seasonal influenza viruses, which require a lower pH of membrane fusion (pH ≤ 5.5) to transmit efficiently by the respiratory airborne route [35]. In this study, we tested the fusion of cattle and goat H5N1 viruses, our data demonstrated syncytium formation at a pH of 5.9, indicating the virus currently has limited potential for human-to-human transmission, an essential pre-requisite to raise the level of pandemic risk. This may also suggest that this virus is currently not spreading efficiently by airborne route between cattle. To support this, although ferrets directly inoculated with the cattle H5N1 virus exhibited noticeable clinical signs and the virus replicated efficiently in the respiratory tract, eyes, and brain. No aerosol transmission was observed from infected ferrets to naïve ferrets [42], a surrogate for human respiratory droplet transmission.

Since the first detection, animal movement has contributed to the virus transmission from farm to farm, however, it is still uncertain how the virus was transmitted between cattle. One potential transmission route among cattle is through the mammary gland. Shared teat orifice and cisternae contaminated with the H5N1 virus could lead to infection of mammary glands and the production of virus-contaminated milk [17]. It has been shown that both avian α-2,3-SA and human α-2,6-SA linkages are distributed in the mammary gland of cattle, suggesting cattle mammary gland could potentially be a novel “mixing vessel” for AIV or be a host in which adaptive mutations towards human receptor binding might be selected [43]. To further test HA adaptations that could potentially increase or switch receptor binding to human-like receptor analogues, we examined the receptor binding profiles of seven emerging mutations, T127A, Q138L, D155N, A156 T, P158Q, I162 V and Q218R, that map close to, or within the receptor binding domain (RBD) of HA. Our data showed that H5 HA mutants bearing any of the seven mutations still exclusively bound the avian-like receptor analogue. Comparing the avidity among the mutants, A156 T introduced an N-linked glycosylation motif, which potentially hinders HA binding to sialic acid and resulted in a 199-fold reduction in receptor binding compared to TX-Cattle WT. This finding is consistent with previous studies on the HA of H7N9 and H9N2, where the introduction of N-linked glycosylation reduced virus binding to sialic acid [29, 31]. In contrast another mutation, Q218R increased receptor binding by 84-fold, the substitution from neutral amino acid glutamine with the positively charged arginine potentially increased virus binding to negatively charged sialic acid due to the charge effect [44]. However, the mechanism by which the virus acquired these mutations requires further investigation. A study has shown that the dairy farm worker isolates with HA T199I mutation by H3 numbering (T195I by H5 numbering) extended virus binding spectrum to α2,3 SA linkages than predominant H5N1 viruses, including 3′ sialyl Lewis X and α2,3 linked lactosamine glycans [41]. This suggests that the glycomic studies on bovine respiratory tract and mammary gland tissues should be conducted and correlated with bovine H5N1 virus infection to gain a better understanding of how the clade 2.3.4.4b H5N1 virus expanded its host tropism in dairy cattle.

We further tested whether there was any change in the required pH for membrane fusion of the mutant viruses. Q138L showed only a slight change of membrane fusion at pH 5.8, while the other 6 mutations retained the WT pH of fusion. Altogether, these findings indicate that despite the presence of α-2,6-SA linkages in cattle, no HA mutations in the cattle H5N1 viruses have yet acquired receptor binding affinity to the human receptor, and the mutant viruses are unlikely to have enhanced human-to-human airborne transmissibility due to retaining a high pH of fusion of 5.8–5.9.

In summary, we conducted a rapid risk profile of the receptor binding properties and fusion pH of “original” dairy cattle, emerging HA mutants in dairy cattle, and goat H5N1 viruses. None of the tested viruses exhibited binding to the human receptor analogue 6SLN and they all retained a high membrane fusion pH ≥5.8, suggesting the currently circulating bovine H5N1 viruses are unlikely to be able to efficiently transmit between humans. However, it remains to be determined whether further HA mutations will emerge if the virus continues to infect and spread amongst dairy cattle, with continuing opportunities to adapt to bovines. Addressing this question will contribute to a better understanding of the H5N1 virus and its adaptation in cattle. Continued surveillance and risk assessment of circulating clade 2.3.4.4b H5N1 viruses remains a top priority to mitigate their potential impact on public health and the agricultural sector.

## Supplementary Material

Supplementary file.docx

## References

[1] Webster RG, et al. Evolution and ecology of influenza A viruses. Microbiol Rev. 1992;56(1):152–179. doi:10.1128/mr.56.1.152-179.1992 1579108 PMC372859

[2] Fereidouni S, et al. Genetic characterization of a new candidate hemagglutinin subtype of influenza A viruses. Emerg Microbes Infect. 2023;12(2):2225645. doi:10.1080/22221751.2023.2225645 37335000 PMC10308872

[3] Li Y, et al. Outbreaks of highly pathogenic avian influenza (H5N6) virus subclade 2.3. 4.4 h in swans, xinjiang, western China, 2020. Emerg Infect Dis. 2020;26(12):2956–2960. doi:10.3201/eid2612.201201 33030424 PMC7706961

[4] group, W.O.F.H.N.e.w. Continued evolution of highly pathogenic avian influenza A (H5N1): updated nomenclature. Influenza Other Resp Viruses. 2012;6(1):1–5. doi:10.1111/j.1750-2659.2011.00298.x PMC5074649 22035148

[5] Cui P, et al. Global dissemination of H5N1 influenza viruses bearing the clade 2.3.4.4b HA gene and biologic analysis of the ones detected in China. Emerg Microbes Infect. 2022;11(1):1693–1704. doi:10.1080/22221751.2022.2088407 35699072 PMC9246030

[6] Bordes, L., et al., Highly pathogenic avian influenza H5N1 virus infections in wild Red foxes (vulpes vulpes) show neurotropism and adaptive virus mutations. Microbiol Spectr, 2023. 11:e02867-22. doi:10.1128/spectrum.02867-22 PMC9927208 36688676

[7] Leguia, M., et al., Highly pathogenic avian influenza A (H5N1) in marine mammals and seabirds in Peru. Nat Commun, 2023. 14(1):5489. doi:10.1038/s41467-023-41182-0 37679333 PMC10484921

[8] Lindh E, et al. Highly pathogenic avian influenza A(H5N1) virus infection on multiple fur farms in the south and central ostrobothnia regions of Finland, July 2023. Euro Surveill. 2023;28(31):2300400. doi:10.2807/1560-7917.ES.2023.28.31.2300400 PMC10401912 37535475

[9] Domanska-Blicharz K, et al. Outbreak of highly pathogenic avian influenza A(H5N1) clade 2.3.4.4b virus in cats, Poland, June to July 2023. Euro Surveill. 2023;28(31):2300366. doi:10.2807/1560-7917.ES.2023.28.31.2300366 PMC10401911 37535474

[10] World Health Organization. Human infection with avian influenza A(H5) viruses. 2024 [cited 2024 2 August 2024].

[11] Callahan D. Stevens County goat tests positive for same influenza virus affecting poultry. 2024 [cited 2024 13th August].

[12] USDA. Federal and State Veterinary, Public Health Agencies Share Update on HPAI Detection in Kansas, Texas Dairy Herds. 2024.

[13] Uyeki TM, et al. Highly pathogenic avian influenza A(H5N1) virus infection in a dairy farm worker. N Engl J Med. 2024;390(21):2028–2029. doi:10.1056/NEJMc2405371 38700506

[14] Le Sage V, et al. Persistence of influenza H5N1 and H1N1 viruses in unpasteurized milk on milking unit surfaces. Emerg Infect Dis. 2024;30(8):1721–1723. doi:10.3201/eid3008.240775 38914418 PMC11286056

[15] Nguyen T-Q, et al. Emergence and interstate spread of highly pathogenic avian influenza A (H5N1) in dairy cattle. 2024; bioRxiv: 2024.05. 01.591751. 10.1126/science.adq0900 40273240

[16] Halwe, N.J., et al. Outcome of H5N1 clade 2.3.4.4b virus infection in calves and lactating cows. 2024.

[17] Caserta LC, et al. (2024). Spillover of highly pathogenic avian influenza H5N1 virus to dairy cattle. Nature. 2024;634:669–676. doi:10.1038/s41586-024-07849-4 PMC11485258 39053575

[18] Hu X, et al. Highly Pathogenic Avian Influenza A (H5N1) clade 2.3. 4.4 b Virus detected in dairy cattle. 2024; bioRxiv: 2024.04. 16.588916.

[19] Skehel JJ, Wiley DC. Receptor binding and membrane fusion in virus entry: the influenza hemagglutinin. Annu Rev Biochem. 2000;69:531–569. doi:10.1146/annurev.biochem.69.1.531 10966468

[20] Gamblin SJ, Skehel JJ. Influenza hemagglutinin and neuraminidase membrane glycoproteins. J Biol Chem. 2010;285(37):28403–28409. doi:10.1074/jbc.R110.129809 20538598 PMC2937864

[21] Rogers GN, Paulson JC. Receptor determinants of human and animal influenza virus isolates: differences in receptor specificity of the H3 hemagglutinin based on species of origin. Virology. 1983;127(2):361–373. doi:10.1016/0042-6822(83)90150-2 6868370

[22] Vines A, et al. The role of influenza A virus hemagglutinin residues 226 and 228 in receptor specificity and host range restriction. J Virol. 1998;72(9):7626–7631. doi:10.1128/JVI.72.9.7626-7631.1998 9696865 PMC110023

[23] Louten, J. Chapter 4 - Virus replication. In: Louten J, editor. Essential human virology. Boston: Academic Press; 2016. p. 49–70. doi:10.1016/B978-0-12-800947-5.00004-1

[24] Russell CJ, Hu M, Okda FA. Influenza hemagglutinin protein stability, activation, and pandemic risk. Trends Microbiol. 2018;26(10):841–853. doi:10.1016/j.tim.2018.03.005 29681430 PMC6150828

[25] Linster M, et al. Identification, characterization, and natural selection of mutations driving airborne transmission of A/H5N1 virus. Cell. 2014;157(2):329–339. doi:10.1016/j.cell.2014.02.040 24725402 PMC4003409

[26] Neumann G, et al. Generation of influenza A viruses entirely from cloned cDNAs. Proc Natl Acad Sci USA. 1999;96(16):9345–9350. doi:10.1073/pnas.96.16.9345 10430945 PMC17785

[27] Lin YP, et al. Evolution of the receptor binding properties of the influenza A(H3N2) hemagglutinin. Proc Natl Acad Sci USA. 2012;109(52):21474–21479. doi:10.1073/pnas.1218841110 23236176 PMC3535595

[28] Xiong X, et al. Receptor binding by a ferret-transmissible H5 avian influenza virus. Nature. 2013;497(7449):392–396. doi:10.1038/nature12144 23615615

[29] Sealy JE, et al. Adsorptive mutation and N-linked glycosylation modulate influenza virus antigenicity and fitness. Emerg Microbes Infect. 2020;9(1):2622–2631. doi:10.1080/22221751.2020.1850180 33179567 PMC7738305

[30] World Health Organization. Manual for the laboratory diagnosis and virological surveillance of influenza. 2011.

[31] Chang P, et al. Immune escape adaptive mutations in the H7N9 avian influenza hemagglutinin protein increase virus replication fitness and decrease pandemic potential. J Virol. 2020;94. doi:10.1128/jvi.00216-20 PMC7495387 32699084

[32] European Food Safety Authority, et al. Avian influenza overview April–June 2023. EFSA Journal. 2023;21(7):e08191. doi:10.2903/j.efsa.2023.8191 37485254 PMC10358191

[33] Byrne AMP, et al. Investigating the genetic diversity of H5 avian influenza viruses in the United Kingdom from 2020-2022. Microbiol Spectr. 2023;11:e04776-22. doi:10.1128/spectrum.04776-22 37358418 PMC10433820

[34] Yang, J., et al. The Haemagglutinin Genes of the UK Clade 2.3. 4.4 b H5N1 Avian Influenza Viruses from 2020 to 2022 Retain Strong Avian Phenotype; 2024.

[35] Peacock TP, et al. Variability in H9N2 haemagglutinin receptor-binding preference and the pH of fusion. Emerg Microbes Infect. 2017;6(1)1–7. doi:10.1038/emi.2016.139 PMC5378918 28325922

[36] Xiong X, et al. Enhanced human receptor binding by H5 haemagglutinins. Virology. 2014;456–457(100):179–187. doi:10.1016/j.virol.2014.03.008 PMC4053833 24889237

[37] Ríos Carrasco M, et al. The mammary glands of cows abundantly display receptors for circulating avian H5 viruses. 2024; bioRxiv: 2024.05. 24.595667. 10.1128/jvi.01052-24 PMC11575340 39387556

[38] Yang G, Ojha CR, Russell CJ. Relationship between hemagglutinin stability and influenza virus persistence after exposure to low pH or supraphysiological heating. PLoS Pathog. 2021;17(9):e1009910. doi:10.1371/journal.ppat.1009910 34478484 PMC8445419

[39] Santos JJ, et al. Bovine H5N1 influenza virus binds poorly to human-type sialic acid receptors. 2024; bioRxiv: 2024.08. 01.606177.

[40] Chopra P, et al. Receptor Binding Specificity of a Bovine A (H5N1) Influenza Virus. 2024; bioRxiv: 2024.07.30.605893.

[41] Good MR, et al. A single mutation in dairy cow-associated H5N1 viruses increases receptor binding breadth. 2024; bioRxiv: 2024.06.22.600211. 10.1038/s41467-024-54934-3 PMC11685663 39737954

[42] Eisfeld AJ, et al. Pathogenicity and transmissibility of bovine H5N1 influenza virus. Nature. 2024;633(8029):426–432. doi:10.1038/s41586-024-07766-6 PMC11390473 38977017

[43] Kristensen C, et al. Avian and human influenza A virus receptors in bovine mammary gland. Emerg Infect Dis. 2024;30(9):1907–1911. doi:10.3201/eid3009.240696 PMC11347012 39127127

[44] Karunarathna TK, et al. Investigation of H9N2 avian influenza immune escape mutant that lacks haemagglutination activity. 2023; bioRxiv: 2023.10. 03.558847.

