# Supplementary material for: The haemagglutinin gene of bovine-origin H5N1 influenza viruses currently retains receptor-binding and pH-fusion characteristics of avian host phenotype
Source: Emerg Microbes Infect. 2025 Jan 13;14(1):2451052. doi: 10.1080/22221751.2025.2451052 (PMC11776067; doi:10.1080/22221751.2025.2451052)
Supplement: Supplementary file.docx [file TEMI_A_2451052_SM9304.docx]

Table S1. Authors, originating and submitting laboratories of the sequences from GISAID used in this study.

| **Segment ID** | **Segment** | **Country** | **Collection date** | **Isolate-ID** | **Isolate name** | **Originating and Submitting Lab** | **Authors** |
| --- | --- | --- | --- | --- | --- | --- | --- |
| EPI1963392 | HA | United Kingdom | 2021-Nov-01 | EPI1963392 | A/chicken/Scotland/054477/2021 | Animal and Plant Health Agency (APHA) |  |
| EPI2089022 | HA | United Kingdom | 2022-Jun-15 | EPI2089022 | A/chicken/England/085598/2022 | Animal and Plant Health Agency (APHA) |  |
| EPI3158678 | HA | United States | 2024-Mar-20 | EPI3158678 | A/dairy cow/Texas/24-008749-001/2024 | National Veterinary Services Laboratories - USDA | Aufderhar,Matthew;  Franzen,Kerrie;Love,Emily;  Killian,Mary;  Lantz,Kristina;Stuber,Tod;  Hicks,Jessica;  Norris,Cameron |
| EPI3162317 | HA | United States | 2024-Mar-08 | EPI3162317 | A/goat/Minnesota/24-007234-003/2024 | National Veterinary Services Laboratories - USDA | Aufderhar,Matthew;  Franzen,Kerrie;Love,Emily;  Killian,Mary;  Lantz,Kristina;Stuber,Tod;  Hicks,Jessica;  Norris,Cameron |

Table S2. HA amino acid differences among the H5N1 viruses used in this study.

| Virus | HA Amino acid difference (H5 numbering) | | | | | | | | |
| --- | --- | --- | --- | --- | --- | --- | --- | --- | --- |
|  | 15 | 71 | 104 | 115 | 195 | 210 | 501 | 508 | 530 |
| TX-Cattle | Q | I | M | Q | I | A | S | V | M |
| MN-Goat | Q | I | M | Q | I | A | R | V | M |
| Sct477/21 | K | I | L | L | T | V | S | I | I |
| Eng598/22 | Q | T | L | L | T | V | S | I | I |

Table S3 HA molecular characteristics of selected H5N1 viruses.

| **Amino acid (H3 numbering)** | **Amino acid (H5 numbering)** | **Sct477/21** | **Eng598/22** | **TX-Cattle** | **MN-Goat** | **Phenotype change** |
| --- | --- | --- | --- | --- | --- | --- |
| H110Y | H103Y | H | H | H | H | Increased HA stability and transmissibility in ferrets |
| S137A | S133A | A | A | A | A | Increased recognition of α-2,6-SA by glycan array |
| G158N | G154N | N | N | N | N | Increased replication in mammalian cells |
| T160A | T156A | A | A | A | A | Increased binding to α-2,3-SA and α-2,6-SA |
| T192I | T188I | T | T | T | T | Increased recognition of α-2,6-SA by glycan array |
| Q192R | Q196R | K | K | K | K | Increased binding to α-2,6-SA |
| Q192H | Q196H | K | K | K | K | Increased binding to human lower respiratory tract |
| N193K | N197K | N | N | N | N | Increased binding to α-2,6-SA |
| W225G | W221G | G | G | G | G | Increased virulence in mice |
| Q226L | Q222L | Q | Q | Q | Q | Increased binding to α-2,6-SA, Increased transmissibility in ferrets |
| G228S | G226S | G | G | G | G | Increased binding to α-2,6-SA, Increased transmissibility in ferrets |


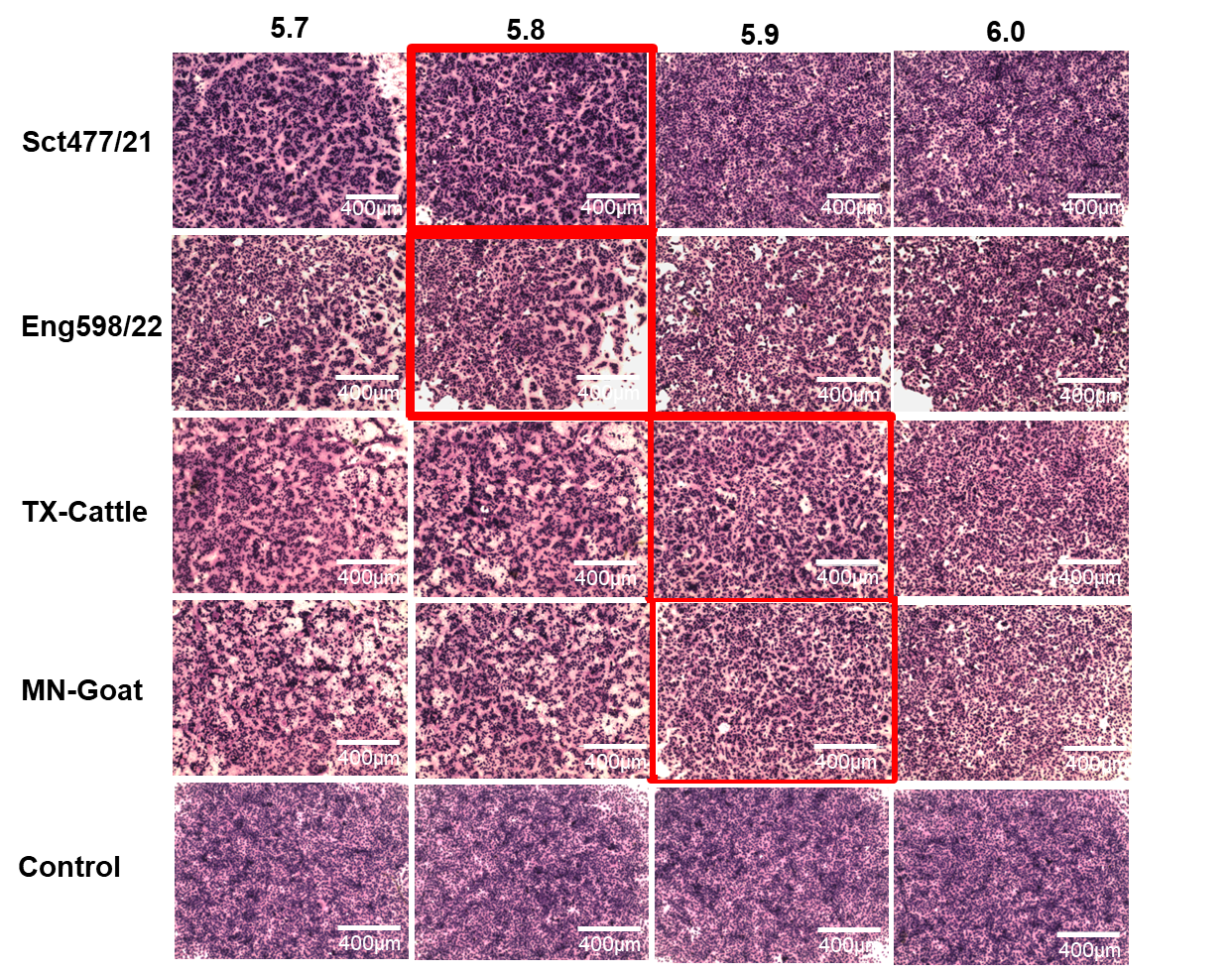


**Figure S1. pH fusion of the panel H5N1 viruses.** Monolayered Vero cells were infected with the panel viruses and subsequently treated with PBS at the indicated pH. Cells were fixed in acetone:methanol (1:1) and stained with Giemsa solution. Images were captured at a scale of 400 µm.


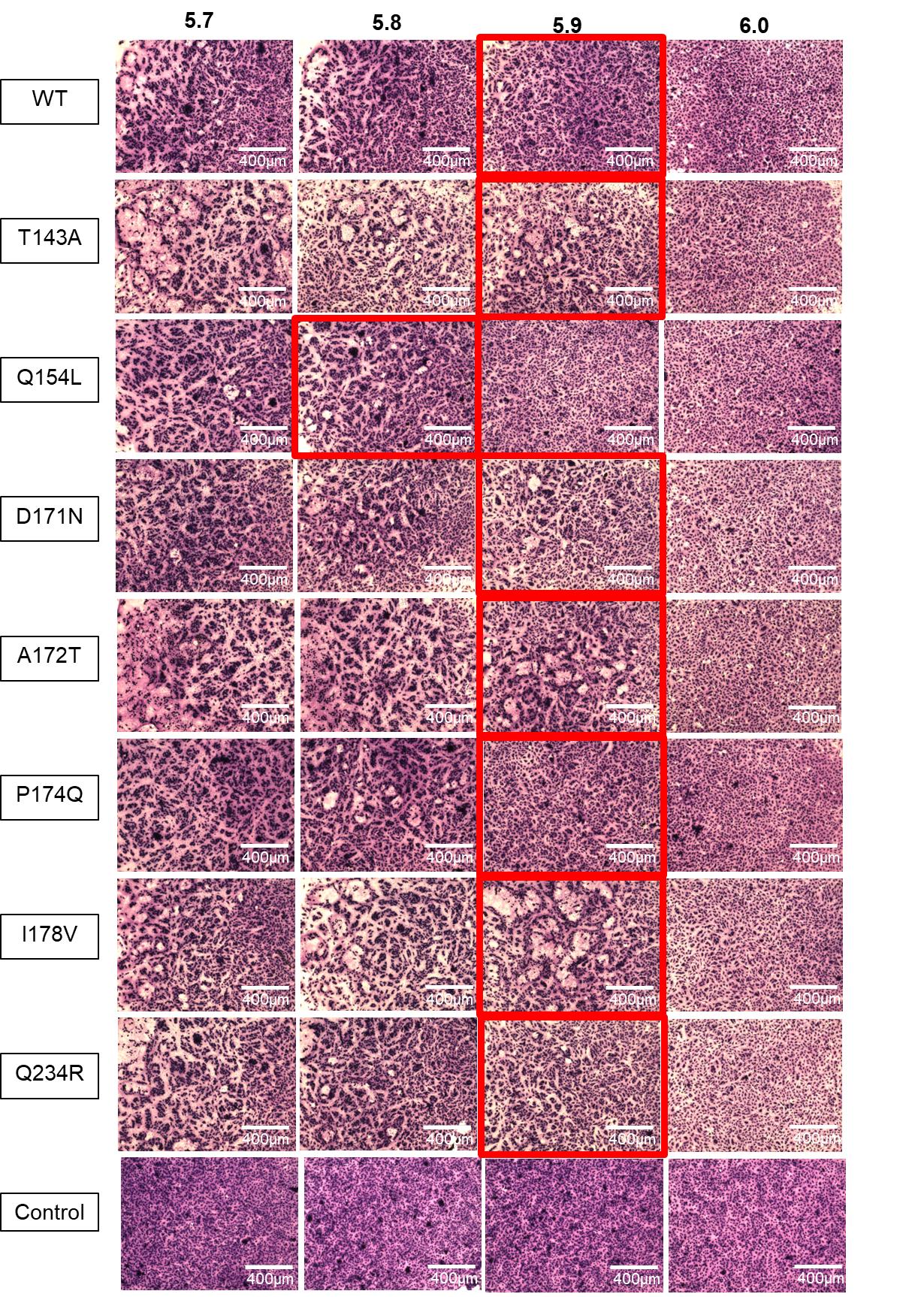


**Figure S2. pH fusion of the H5N1 mutant viruses.** Monolayered Vero cells were infected with the mutant viruses and subsequently treated with PBS at the indicated pH. Cells were fixed in acetone:methanol (1:1) and stained with Giemsa solution. Images were captured at a scale of 400 µm.
